# A synthesis of recent analyses of human resources for health requirements and labour market dynamics in high-income OECD countries

**DOI:** 10.1186/s12960-016-0155-2

**Published:** 2016-09-29

**Authors:** Gail Tomblin Murphy, Stephen Birch, Adrian MacKenzie, Stephanie Bradish, Annette Elliott Rose

**Affiliations:** 1 Dalhousie University, 5869 University Avenue, Halifax, NS B3H 4R2 Canada; 2 McMaster University, 1280 Main Street West, Hamilton, ON L8S 4K1 Canada

**Keywords:** HRH planning, Health workforce planning, Health workforce requirements, OECD countries, High-income countries

## Abstract

**Background:**

Recognition of the importance of effective human resources for health (HRH) planning is evident in efforts by the World Health Organization (WHO) and the Global Health Workforce Alliance (GHWA) to facilitate, with partner organizations, the development of a global HRH strategy for the period 2016–2030. As part of efforts to inform the development of this strategy, the aims of this study, the first of a pair, were (a) to conduct a rapid review of recent analyses of HRH requirements and labour market dynamics in high-income countries who are members of the Organisation for Economic Co-operation and Development (OECD) and (b) to identify a methodology to determine future HRH requirements for these countries.

**Methods:**

A systematic search of peer-reviewed literature, targeted website searches, and multi-stage reference mining were conducted. To supplement these efforts, an international Advisory Group provided additional potentially relevant documents. All documents were assessed against predefined inclusion criteria and reviewed using a standardized data extraction tool.

**Results:**

In total, 224 documents were included in the review. The HRH supply in the included countries is generally expected to grow, but it is not clear whether that growth will be adequate to meet health care system objectives in the future. Several recurring themes regarding factors of importance in HRH planning were evident across the documents reviewed, such as aging populations and health workforces as well as changes in disease patterns, models of care delivery, scopes of practice, and technologies in health care. However, the most common HRH planning approaches found through the review do not account for most of these factors.

**Conclusions:**

The current evidence base on HRH labour markets in high-income OECD countries, although large and growing, does not provide a clear picture of the expected future HRH situation in these countries. Rather than HRH planning methods and analyses being guided by explicit HRH policy questions, most of the reviewed studies appeared to derive HRH policy questions based on predetermined planning methods. Informed by the findings of this review, a methodology to estimate future HRH requirements for these countries is described.

**Electronic supplementary material:**

The online version of this article (doi:10.1186/s12960-016-0155-2) contains supplementary material, which is available to authorized users.

## Background

The backbone of any health care system is the human resources who deliver care. Thus, human resources for health (HRH) planning has a direct impact on the functioning of health care systems, which are critical to ensuring a healthy population. According to one of the early seminal texts on the subject, HRH planning is,

the process of estimating the number of persons and the kind of knowledge, skills, and attitudes they need to achieve predetermined health targets and ultimately health status objectives. Such planning also involves specifying who is going to do what, when, where, how, and with what resources for what population groups or individuals so that the knowledge and skills necessary for the adequate performance can be made available according to predetermined policies and time schedules. This planning must be a continuing and not a sporadic process, and it requires continuous monitoring and evaluation. [1].

Put another way, it involves matching the supply of HRH to the requirements for the services they provide [2]. Despite the centrality of HRH planning to the success of global campaigns such as the Millennium Development Goals [3], it remains a significant challenge. For example, many countries still lack the capacity to maintain accurate counts of their health care providers [4]. Other countries’ efforts focus on monitoring HRH supply without considering whether it is adequate to meet HRH requirements [5, 6].

Recognition of the importance of effective HRH planning is evident in efforts by the World Health Organization (WHO) and the Global Health Workforce Alliance (GHWA) to facilitate, with partner organizations, the development of a global HRH strategy for the period 2016–2030 [7]. These efforts build on a number of activities undertaken by the WHO to promote evidence-based HRH planning among member countries. For example, the HRH Observer series, published by WHO, has included technical issues on tools for modelling HRH supply and requirements [8], measuring inequalities in the distribution of HRH [9], and analysing HRH labour markets in developing countries [10], among other topics relevant to HRH planning.

To inform this process, the HRH unit of the WHO requested that the authors first conduct a rapid review of recent analyses of HRH requirements and labour market dynamics in the member countries of the Organisation for Economic Co-operation and Development (OECD) who are classified as ‘high income’ by the World Bank. These include Australia, Austria, Belgium, Canada, Chile, the Czech Republic, Denmark, Estonia, Finland, France, Germany, Greece, Hungary, Iceland, Ireland, Israel, Italy, Japan, Luxembourg, the Netherlands, New Zealand, Norway, Poland, Portugal, Slovakia, Slovenia, Spain, Sweden, Switzerland, the United Kingdom, and the United States (referred to hereafter as the ‘included countries’). Second, it was requested that the authors identify, informed by the findings of the review, a methodology to determine future HRH requirements for these countries.

The specific objectives of the review were as follows:

(I) To identify all analyses of HRH requirements and health care labour market dynamics for high-income OECD countries published in English within the past 10 years
(II) To categorize the analyses according to the type(s) of models used to estimate requirements, the professions included, the time frames over which they apply, any labour market trends identified, and any assumptions on which they were based
(III) To identify key themes and trends in these analyses emerging over time
(IV)     To identify and report gaps in the knowledge base formed by these analyses to inform the development of a global HRH strategy
(V)   To identify a methodology to estimate future HRH requirements in OECD countries

## Methods

Multiple steps were taken to obtain all possible relevant analyses of HRH requirements and labour market dynamics used in the last decade for the included countries. These included a systematic search of published peer-reviewed literature, targeted searches of key HRH websites, and multi-stage mining of the references of documents obtained through these means. This analysis was undertaken in collaboration with colleagues in the WHO’s Health Workforce unit, who contributed to the development of the search parameters, helped identify potentially relevant websites, and invited prospective members to join an international Advisory Group (AG) of HRH researchers.

The search of the peer-reviewed literature targeted several electronic databases, including PubMed, Informa HealthCare, Web of Science, EconLit, ABI/INFORM, and CINAHL. These were searched for articles whose titles or abstracts contained terms from each of two groups:

1. Any of the following: Health human resources, HHR, human resources for health, HRH, health workforce, health workers, health manpower, doctors, physicians, nurses, midwives, pharmacists, dentists AND
2. Any of the following: planning, forecasting, modeling, requirements, needs, demand, gaps, shortage, supply, oversupply, labor market, dynamics, horizon scan, Australia, Austria, Belgium, Canada, Chile, Czech Republic, Denmark, Estonia, Finland, France, Germany, Greece, Hungary, Iceland, Ireland, Israel, Italy, Japan, South Korea, Luxembourg, the Netherlands, New Zealand, Norway, Poland, Portugal, Slovakia, Slovenia, Spain, Sweden, Switzerland, UK, USA, OECD, high-income

Websites of multiple national organizations such as departments of health, bureaus of statistics, and HRH-specific planning bodies were searched, as well as those of international organizations such as the WHO, the GHWA, the OECD, and the World Bank. When search functions were available for website content, the relevant search terms presented above were used. If search functions were not available, sections of these websites titled ‘Publications’, ‘Reports’, or similar were searched for documents pertaining to HRH analyses. A full list of the websites searched is included as Additional file 1.

The database and website searches yielded over 1000 documents deemed to be potentially relevant. The titles, abstracts, and/or executive summaries of these documents were then reviewed to ensure that they were published in English between March 2005 and March 2015 and included a primary analysis of HRH requirements or labour market dynamics in one or more included countries.

**Fig. 1:**
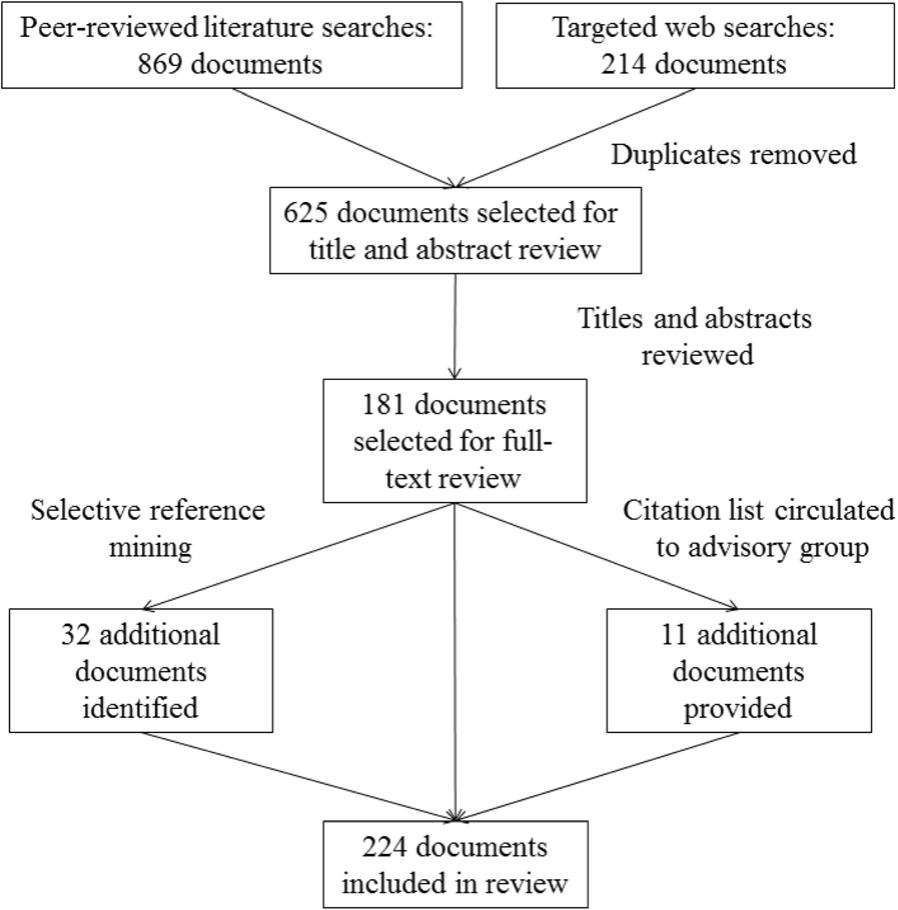
Search strategy

Based on these criteria, 181 documents were selected for full-text review. Although it was not possible within the allotted time to mine the reference lists of all 181 documents, a selection of the 30 documents that cited the most potentially relevant works were mined for additional documents. The initial list of documents resulting from these activities was then circulated to the AG with the request that they identify any relevant works they felt had not yet been included. The search process is illustrated in Fig. 1.

In total, 224 documents were included in the review. A data extraction tool was created to facilitate summarization of key features of each of these documents in terms of their scopes, methods, conclusions, and any major issues or themes identified. A copy of this tool is available as Additional file 2.

## Results

### Jurisdictional focus

**Fig. 2:**
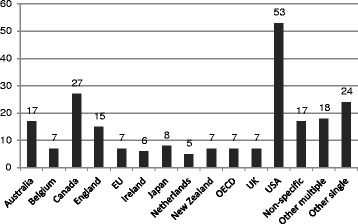
Number of documents by jurisdiction of focus

The documents reviewed covered the 32 included countries specifically, with others looking at groups of countries (such as the European Union or WHO regions) or globally (Fig. 2). The United States was the jurisdiction covered by the most documents, with 53 (24 % of all documents) specific to that country, followed by Canada with 27 (12 %). Thirty-two documents (14 %) focused on multiple countries, most commonly the European Union and the OECD with seven documents (3 %) each. Most of the documents focusing on individual countries were those where English is an official language—not surprising given the search parameters—although it is noteworthy that Belgium, Japan, and the Netherlands were each the focus of at least five documents. Other countries for which more than one document was found included Finland (seven), Germany (three), Iceland (two), Israel (two), Italy (two), Norway (three), Portugal (two), Scotland (four), and Wales (two).

### Professions included

**Fig. 3:**
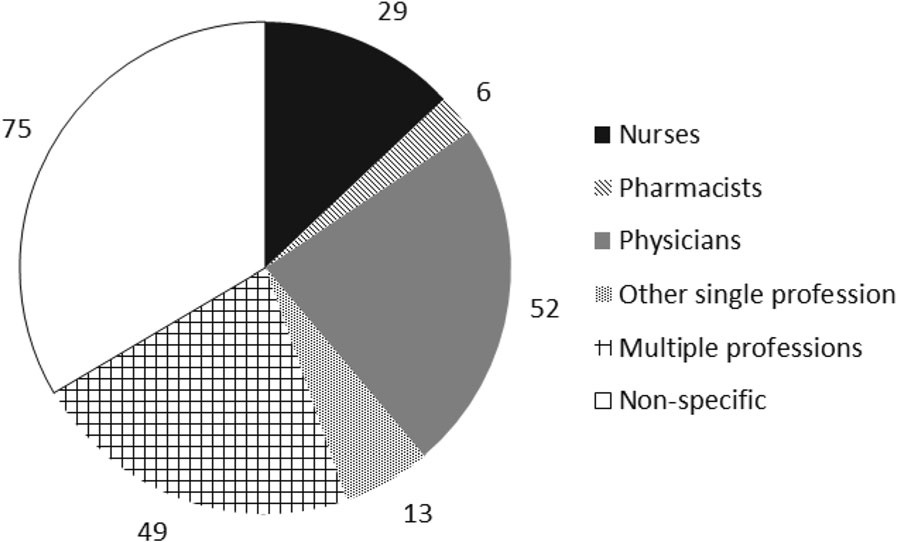
Number of documents by profession of focus

A diverse range of health professions were covered in the documents (Fig. 3). Seventy-four documents were not specific to any particular health profession, instead analysing health care system and labour market issues that affect all health professions to varying degrees. Among documents focusing on a single profession, 52 (23 %) documents covered physicians, 29 (13 %) covered nurses, and pharmacists were covered in 6 (3 %) documents. The only other professions to be the sole focus of more than one document were dentists (four), midwives (two), and physiotherapists (two).

Although 49 documents included analyses for more than one profession, the majority of these (38) considered those professions in isolation of each other. Only 11 of these included analyses that considered the interdependence between different professions.

### Date of publication

**Fig. 4:**
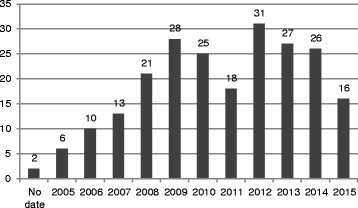
Number of documents by year of publication

Although the review spanned the past decade, 144 documents (64 % of those included) were published in 2010 or later (Fig. 4). The number of documents published increased annually from 2005 through 2009, with the maximum of 32 published in 2012.

### Analytical time frames

**Fig. 5:**
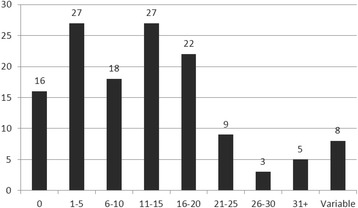
Number of documents by analytical time frame

The documents that provided quantitative analyses of HRH supplies or requirements did so over varying time periods (Fig. 5). Sixteen analyses were cross-sectional, providing HRH analyses for one particular point in time. The others varied widely in the length of time they covered, with 27 documents each including projections 1 to 5 years ahead and 11 to 15 years ahead. Five documents included projections more than 30 years into the future, while eight used multiple projection periods in their analyses.

### Types of models used

**Fig. 6:**
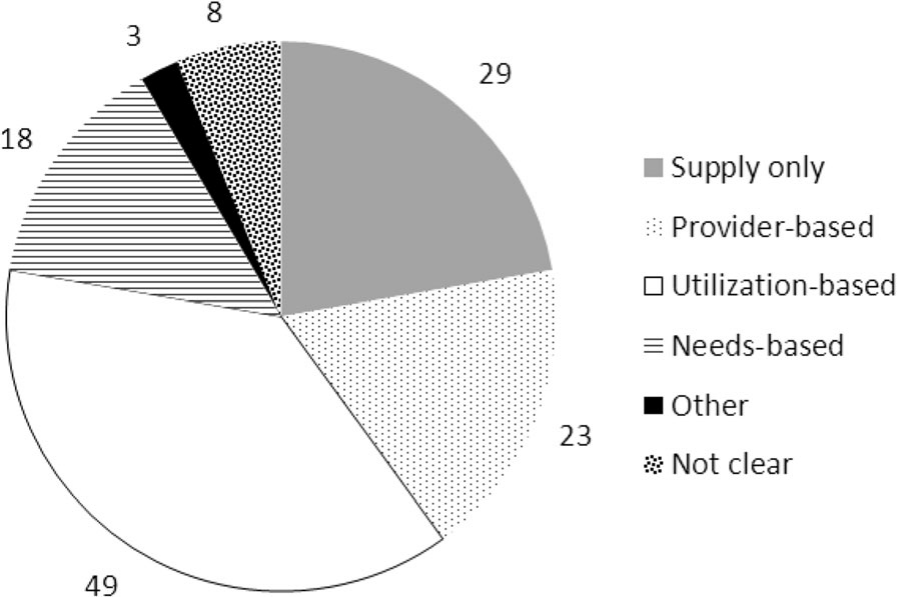
Number of documents by type of model used

A number of different types of models were used to perform the HRH analyses in the included documents, which we have grouped into three categories (Fig. 6). These include provider-based (sometimes referred to as supply-based), utilization-based (sometimes referred to as demand-based), and needs-based approaches. Utilization-based approaches are those in which current or target rates of health service utilization are multiplied by estimates of future population size, which are then converted to HRH requirements using productivity estimates. Provider-based approaches are those in which HRH requirements are estimated primarily by multiplying current or target provider-population ratios to the estimated size of the future population, sometimes adjusting for basic demographic factors like age and sex. Needs-based approaches are those that determine HRH requirements by applying estimated future levels of health in the population to best practices (or current policy) for service provision in response to different levels of health. Provider requirements are then estimated from the best practice (or current productivity norms) of delivering care to meet those health care needs. It should be noted that the categorization of models in Fig. 6 is based on their characteristics and this typology as opposed to how authors’ document may have characterized them (see the ‘Results’ section for more detail on the different use of terms such as ‘need’ and ‘demand’ across documents).

Of the 224 documents reviewed, 130 described quantitative analytical methods and/or analyses of HRH supply or requirements (as opposed to, for example, focusing on a qualitative discussion of HRH labour market or policy issues). Of these, 29 (22 %) analysed HRH supply only—i.e. without contrasting it with HRH requirements. Among those documents analysing requirements, 49 (38 %) used utilization-based approaches. Twenty-three (18 %) used provider-based approaches while 18 (14 %) used needs-based approaches. We were not able to determine which type of approach was used in eight documents (6 %).

The approaches described in three documents (2 %) did not fit any of these categories. The primary determinants of HRH requirements described in these documents included the following:

- ‘Local health board plans’ [11]
- Health needs, willingness/ability to pay, regulatory structures [12]
- Demography, health needs, service levels, productivity, ‘clinical care microsystems’, ‘policy/governance/power’, ‘funds and support’, ‘individual response:behaviour biology’—several of these are identified as influencing both HRH supply and requirements [13]

### Assumptions described

A detailed analysis of the various assumptions used in each of these planning models is difficult because such assumptions are often implicit. However, the assumption most commonly articulated in these documents was that ‘the status quo will continue’—generally that the only model parameters whose value will change over time are those pertaining to population demographics, while other parameters such as service delivery models, practice patterns, productivity, and prevalence rates of various health conditions are assumed to remain constant. Three other explicit assumptions noted in multiple documents were as follows:

- There are no unmet HRH requirements in the base (or first) year of the analysis (see, for example, [14]).
- Utilization of services is a proxy for demand (see, for example, [15]).
- The current ratio of HRH to population is adequate (see, for example, [16]).

Although the assumptions used in these analyses were often not stated explicitly, 13 documents (6 %) included multiple scenarios to demonstrate the sensitivity of their results to different parameter values such as HRH retirements, population growth, or productivity. Each of these demonstrated that projections of future HRH supply and requirements are highly sensitive to different assumptions about how various determinants would change over time.

### Trends and issues identified

A number of trends and issues were raised repeatedly in these documents, many of which have been identified by other recent reviews of HRH studies (e.g. [5, 6, 17, 18]). An exhaustive list of citations for documents in which each issue is raised would be prohibitively long, but for each of these recurring topics, a few relevant documents are cited as examples. These recurring topics are listed below under two additional subheadings: ‘Inputs into HRH planning’ and ‘HRH planning methods’.

#### Inputs into HRH planning

##### Aging populations

The most frequently raised issue across these documents was the aging of populations. For example, the population aged 65 and older across all OECD countries is expected to increase by over 70 % from 208 million in 2015 to 355 million in 2050, while the number of those aged 80 and over is expected to increase from 56 million to 133 million over the same period [19]. Most of the reviewed documents indicate that this growth in older populations is expected to significantly increase requirements for health care in these countries, because older people tend to use more health care. Other reviewed documents, however, note that this view conflates the relationships between aging, population health, and service requirements They explained that, while the probability of sickness increases with age, population aging is partially reflective of improvements of the average level of health within each age group, and as such, the failure to account for this distinction between demographic and epidemiological shifts results in overestimates of HRH requirements [20–24].

##### Chronic disease and comorbidities

Multiple documents identified a trend of increasing prevalence of multiple chronic conditions as likely to contribute to increased future requirements for HRH (e.g. [25, 26]). Only a small minority of these, however, included empirical analyses of such trends and their potential impacts on HRH requirements (e.g. [27]). Others emphasize that this change in the complexity of health care needs will require changes in the skill mix of available HRH (e.g. [28]) and/or the models of care delivery they use (e.g. [5, 6, 29]); however, we found no documents that modelled the impacts of changes in needs and potential associated changes in skill mix or care models in terms of their impacts on HRH requirements.

##### Aging workforces

Many of the included documents reported that the stock of available HRH in the jurisdictions to which they pertained is aging, for example, an average of one in three physicians in OECD countries is aged 55 or older [28]. This has prompted concerns about large future HRH shortages, and a number of documents included specific analyses of the potential impacts of different retirement scenarios on HRH supply in their respective jurisdictions (e.g. [29]). In some documents, it is suggested that strategies to encourage HRH to delay retirement be considered as a means of addressing HRH shortages (e.g. [30]).

##### HRH migration

HRH migration across countries is frequently raised as an issue in these documents and in several cases is the primary focus of the analysis (e.g. [31, 32]). Perspectives on migration differ across documents, for example, migration is variously identified as a leading cause of (e.g. [33–35, 36]) or important solution to (e.g. [37, 38]) existing and/or projected future imbalances. Others, in contrast, examine the potential impacts of reducing in-migration from other countries, for example, due to concerns about self-sufficiency and the ethics of international recruitment of HRH (e.g. [27, 39]) or in response to perceived HRH surpluses (e.g. [40]).

##### Distribution of resources

The planning models included in the review focus on optimizing the number of HRH at a particular jurisdictional level; ensuring those resources are appropriately distributed within a given jurisdiction is an important but separate issue. Regardless of the methods used to analyse HRH requirements, many documents noted that estimated surpluses or shortages at one jurisdictional level (e.g. national) may mask shortages or surpluses at other (e.g. state or municipal) levels (e.g. [25, 41]). Several types of HRH imbalances are discussed. Most often, the imbalance noted is between urban and rural areas (e.g. [34, 42–44]). Other types of imbalances described are those that occur across professions, sectors, and/or specialties, such as between the acute and primary care sectors (e.g. [16, 45–48]).

##### Interprofessional education and practice

A number of documents included in this review have identified that modern health care increasingly requires the competencies of more than one profession so that systems must begin to rely increasingly on multi-professional teams of health care providers [5, 49–51]. As such, they note the increasing importance of HRH planning models with the capacity for team-based planning as opposed to single professions in isolation (e.g. [5, 6, 16, 47, 52]). However, only a few such models—from Australia [53–56], Canada [57, 58], and New Zealand [29, 59–61]—were found through this review.

##### Changing care delivery models

Although most of the approaches reviewed assume that current care delivery models will continue in the future, a number of documents (e.g. [28, 62–64]) identify a need for HRH planning models that can incorporate or accommodate changes in care delivery models which may be required to address changes in health care needs and/or address those needs in more effective or efficient ways. Comparatively few of the approaches reviewed [29, 53–61] appear to have the capacity to account for such changes.

##### Changing practice patterns

Changing practice patterns or other provider behaviours are identified in some documents as determinants of HRH requirements. A frequently cited example of such a change is a reduction in the working hours of physicians; other things equal, such reductions reduce the effective supply of physicians and, by extension, the supply of the services they provide. Although decreases in physician working hours are frequently attributed to the growing proportion of female physicians (e.g. [25, 30, 43, 65–67]), other evidence indicates that this reduction in activity is occurring among all physicians, across gender and age groups [68, 69].

##### Evolving scopes of practice and regulatory structures

The issue of scopes of practice is addressed in several documents, specifically in terms of expanding scopes of different health professions over time. A specific example involves task-shifting, whereby tasks traditionally performed by one type of HRH (usually relatively expensive and/or scarce, e.g. physicians) are instead delegated to another type of HRH (usually less expensive and/or scarce, e.g. nurses) [5]. Other things equal, task-shifting reduces requirements for the former profession(s) while increasing them for the latter—such as the expansion of Nurse Practitioner scopes of practice and the potential reduction in demand for physicians [70]. More broadly, it was noted that the evolving scopes of practice for all types require increasing familiarity with a growing range of technologies [47]. Although some approaches include the capacity to adjust for changes in scopes of practice (e.g. [29, 55]), similar to another recent review of HRH planning approaches [6], we found no examples of such changes actually being accounted for in modelling exercises. In none of the included documents were potentially contracting scopes of practice discussed.

Changes to legislation and the other structures that govern the regulation, management, and delivery of health care are identified as an issue in several documents. An example is the Affordable Care Act (ACA) in the United States; one estimate suggests that the ACA will substantially increase demand for nurses [71]. Other examples of regulatory developments likely to impact HRH labour markets are changes to retirement age in countries such as France [6] and policies aimed at regulating dual practice by physicians [72]. More broadly, it is becoming increasingly recognized that existing national regulatory structures require strengthening to ensure the adoption and implementation of effective HRH policies (e.g. [12, 73, 74]).

##### Incentives

Several documents (e.g. [72, 75–77]) discuss the importance of provider financial and non-financial incentives as determinants of provider behaviour. Some of these emphasize the importance of considering these issues within the broader economic context in which HRH live and work (e.g. [40, 78–80]). However, relatively few documents (e.g. [49, 71, 76, 81–83]) include quantitative analyses of the relationships between these incentives and aspects of HRH supply (including levels of participation and activity) or requirements (e.g. as influenced by productivity rates). Some documents factor provider rates of pay into future HRH supply and requirement estimates (e.g. [84–87]); others explicitly investigate the relationship between different incentives and HRH outcomes such as labour force participation and activity (e.g. [81]). It was also emphasized that the effectiveness of various incentives will vary according to different economic contexts [80].

##### Technological changes

Many of the reviewed documents identified technological change as a factor likely to have a significant impact on HRH balance in their respective jurisdictions (e.g. [5, 64, 88]). These include changes in the technology used in health care—such as those allowing for less invasive surgeries [4]—as well as those used by health care consumers, such as mobile phone apps for the management of chronic conditions (e.g. [89]). The impacts of such changes in terms of HRH, however, are not clear from the analyses included in this review. Comparatively few documents directly incorporate consideration of technology changes into quantitative analyses. Among those that do (e.g. [63, 90]), the exact nature of the changes in question is not made explicit.

##### Balancing the private and public sectors

The health care systems in the included countries incorporate a mix of public and private service providers, and the relative size of these sectors varies considerably across and within countries. The public sector is the focus of the vast majority of the included analyses, although several documents emphasize the need for better data on, and coordination by national planners with, the private health care sectors (e.g. [6, 91, 92]). An example of the challenges in effectively managing both the public and private health care systems was raised in multiple documents (e.g. [72, 82]) discussing the issue of dual-practising clinicians—i.e. those who provide services in both the public and private sectors—noting that policies intended to dissuade them from devoting time to private practice may instead drive them to leave the public sector entirely.

#### HRH planning methods

##### Inconsistent use of terms

Terminology differs across documents, with terms such as ‘need’, ‘demand’, and ‘utilization’ used almost interchangeably by some authors. Crettenden and colleagues [93], for example, use utilization as a measure of demand, suggesting that the authors consider these to be equivalent. Other documents note that utilization represents the intersection of supply and demand and, unlike in most markets, demand for health care is not independent of supply, which can result in market failure [2, 72, 78]. Similarly, the Centre for Workforce Intelligence [15] describes its model as estimating demand but uses utilization to measure demand and identifies a needs-based approach [2] as the basis for the model. Other documents, in contrast, emphasize that these are three distinct constructs, none of which can be considered to be a measure of the other (e.g. [2, 72, 94]).

In addition, the term ‘shortage’ is used to describe different HRH situations in different documents. For example, HRH shortages are defined in terms of vacant HRH positions (e.g. [95, 96]) or measured as the degree to which jurisdictional HRH requirements exceed supply (e.g. [15, 65, 91]). Similarly, the presence of unemployment among HRH is deemed by some to reflect a surplus (e.g. [97]) whereas in others a surplus is considered to mean that the jurisdictional supply is greater than requirements (e.g. [86, 98, 99]). Others depict surpluses or shortages as determined at least in part by opinion (e.g. [96, 100, 101]).

##### Stakeholder engagement

The planning approaches identified through this review include a mix of theoretical and applied methods. Among the latter, the importance of stakeholder engagement is acknowledged to varying degrees. Perhaps the most thoroughly documented examples of stakeholder engagement in HRH planning methods are the elicitation and horizon scanning efforts described by the United Kingdom’s Centre for Workforce Intelligence [102, 103]. As part of this approach, multiple groups of stakeholders are engaged in a variety of activities at different stages of the analytical process to help planners identify current and potential future HRH planning issues, articulate potential policy scenarios, supplement administrative data with professional opinions, and interpret and validate results based on their respective experiences.

##### Data quality

The validity of any HRH planning exercise is contingent upon the availability, relevance, and the accuracy of the data to which planners have access. Needs-based approaches in particular require comprehensive data on not only the supply of HRH but also on the health of the population, planned levels of service provision for different health problems, and HRH productivity, which may not always be available. Despite the online availability of important HRH-related data from organizations such as the WHO and the OECD, almost every document included in this review articulated concerns about inadequacies in the data available to inform HRH planning (e.g. [3, 4, 75, 86]).

##### Iterative planning

Many documents (e.g. [2, 5, 6, 64]) emphasized the importance of HRH planning being conducted on an iterative, ongoing basis. This is not only so that planning models can be updated with the most current available data but also so that the relevant stakeholders can be engaged, the validity of any necessary assumptions can be tested, and the effectiveness of implemented strategies can be regularly assessed.

## Discussion

### Limitations of this review

Despite our efforts to assemble a complete set of HRH analyses pertaining to the included countries, some relevant documents may have been missed for any of the following reasons:

- It was not possible to include documents written in languages other than English. This is a particularly important limitation given that English is not an official language for some countries included in the review.
- Not all potentially relevant websites could be included in the review, and those that were included could not be searched exhaustively.
- It was not possible to mine the references of every relevant document.
- Some relevant peer-reviewed publications may not have been part of the databases searched and/or may not have included any of the search terms used in their titles or abstracts. This may be due to variations in the use of headings and sub-categories in the various databases or in the language used to describe HRH research and planning (see the ‘Results’ section for examples).

### Limitations of the available evidence

Although many documents have been published describing various aspects of HRH markets in included countries, they do not collectively present a clear or consistent picture of what the HRH situation is expected to be across—or even within—these jurisdictions in the future. There are several reasons for this:

- The HRH research and/or policy question(s) to be answered by the various analyses are often not clear; different approaches may be required to answer different questions.
- As shown in Fig. 3, the majority of the available studies focus on physicians and nurses; there is little evidence on the supply of—let alone the requirements for—the other HRH that make up each country’s workforce.
- Many of the analyses identified through this review were specific to sub-jurisdictions of the included countries, for example, to one Canadian province [104] or to England [86] as opposed to the entire United Kingdom. Such analyses cannot provide a complete picture of the overall HRH situations in the countries. For most of the jurisdictions included in this review, no quantitative analyses of national-level HRH gaps—i.e. the difference between HRH supply and HRH requirements—were found. For others, only analyses of HRH supply (not requirements) were found, so it is not possible to present a picture of the HRH situation across the included countries here.
- Even among those analyses that project HRH shortages or surpluses, these are measured in different units—for example, head counts as opposed to full-time equivalents (FTEs) in different studies—they span differing time periods, and are specific to a variety of different sectors (e.g. primary care, mental health services, and long-term care).
- As outlined above, there are important differences in the various approaches used to conceptualize and measure HRH supply, requirements, shortages, and surpluses across these countries. Some of these approaches, for example, describe their approach as measuring HRH requirements in terms of demand, even though demand in the market for health care is not independent of supply [2, 72, 94], while others do so in terms of need, which is independent of supply. Given these two fundamentally different concepts, estimates of HRH ‘demand’ cannot be meaningfully combined with estimates of HRH needs, since they are likely to address significantly different policy questions and hence generate different conclusions about the adequacy of HRH supply relative to requirements.
- Estimates of future HRH supply or requirements, however sophisticated, require numerous assumptions. As a number of the reviewed documents show, the estimated future HRH supplies and requirements are sensitive to differences in these assumptions. Even a few modest changes to these assumptions can mean the difference between a large estimated future shortage and a large estimated future surplus of HRH for a given jurisdiction and time period (e.g. [27, 105]).

To illustrate the implications of the above complexities, the substantial gaps in the available evidence, and the qualitatively and quantitatively different conclusions reached by different HRH analyses, Table 1 summarizes the results of the reviewed documents pertaining to Registered Nurses (RNs). Although several of these documents included multiple scenarios in their estimates, in the interests of a minimal level of comparability, the ‘status quo’ scenario is cited below unless otherwise stated.

**Table 1:** Projected national nurse shortages or surpluses by country for 2025

Country	Document	Projected 2025 shortage (−) or surplus (+)
Australia	Health Workforce Australia, 2012a	−109,400 (includes RNs and ENs)
	Health Workforce Australia, 2012b	−80,142
	Health Workforce Australia, 2014	−85,000 (includes RNs and ENs)
Austria	No gap analyses found	
Belgium	No gap analyses found	
Canada	Tomblin Murphy et al., 2012	−60,000
Chile	No gap analyses found	
Czech Republic	No gap analyses found	
Denmark	No gap analyses found	
Estonia	No gap analyses found	
Finland	No gap analyses found	
France	No gap analyses found	
Germany	Maier and Afentakis, 2013	−195,000 (includes RNs and ENs; estimated from graph)
Greece	No gap analyses found	
Iceland	No gap analyses found	
Ireland	Training and Employment Authority, 2009	−836 (for year 2020)
Israel	No gap analyses found	
Italy	No gap analyses found	
Japan	No gap analyses found	
Luxembourg	No gap analyses found	
Netherlands	No gap analyses found	
New Zealand	No gap analyses found	
Norway	No gap analyses found	
Poland	No gap analyses found	
Portugal	No gap analyses found	
Slovakia	No gap analyses found	
Slovenia	No gap analyses found	
Spain	No gap analyses found	
South Korea	No gap analyses found	
Sweden	No gap analyses found	
Switzerland	No gap analyses found	
United Kingdom	Centre for Workforce Intelligence, 2013	−50,000 (for England only; estimated from graph; taken as midpoint of the range of demand and supply projection scenarios)
United States	Aiken and Cheung, 2008	−1,016,900 (for year 2020; analysis from another study)
	Juraschek et al., 2012	−918,232 (for year 2030)
	HRSA, 2014	+340,000

As shown in Table 1, for the majority of included countries, no estimates of the size of any future RN shortage or surplus was found during this review. For the few jurisdictions for which such estimates were found, studies using different methods reached different conclusions. For example, Juraschek and colleagues [106] projected a shortage of over 900 000 RNs in the United States by 2030; meanwhile, the HRSA [41], using different methods and assumptions, estimated a surplus of over 300 000 RNs for 2025.

A similar table for physicians would contain more information but no more clarity, since most analyses of physician supply and requirements focus on particular specialty areas such as emergency care [107] or obstetrics [108] as opposed to the physician supply as a whole. As Fig. 2 suggests, such a table for other professions would be even more sparsely populated. Virtually all documents reviewed seemed to suggest that HRH supply was expected to increase. Exceptions included, for example, nuclear medicine technologists in Australia [109], surgeons in Japan [66], obstetricians-gynaecologists in the United States [108], and RNs in Israel [110]. In contrast, as exemplified by the United States studies cited above, there are differing views about whether the requirements for different types of HRH are expected to increase or decrease in the future in different jurisdictions. Although most of the included analyses for nurses and physicians suggest that national-level shortages are likely in the next decade, several analyses for other non-physician health professions such as pharmacists and dentists suggest substantial surpluses are likely over this time period (e.g. [111, 112]). Should the scenarios depicted in these projections come to pass, the potential for substitution of different types of HRH would become an increasingly important policy issue; however, as noted above, most of the approaches identified through this review lack the capacity to account for such substitution.

### Knowledge gaps resulting from these limitations

There has been a considerable amount of study devoted to the current and potential future states of the supply of and requirements for HRH in high-income OECD countries. Despite these efforts, however, no clear or consistent picture of the status of these countries’ HRH appears to exist. Some of the gaps in information are likely due to limitations in the various strategies used here to collect HRH analyses for this review. However, other recent studies, which did not have the same limitations (e.g. [6]), describe similar gaps in the information available. This is partly the result of significantly different approaches to HRH planning being used across and within these countries. The appropriateness of different HRH planning approaches for given jurisdictions depends on the objectives of the health care systems and the precise policy questions being asked for which they are planning as well as the context in which that planning takes place.

#### A methodology for projecting HRH requirements in high-income OECD countries

The final objective of this review was to identify a methodology for projecting HRH requirements in high-income OECD countries. To guide the identification of such a methodology, a draft set of evaluation criteria was developed and circulated to the Advisory Group for their input, which was considered for the development of the list below:

1. The approach is consistent with the objectives of the health care system.
  This means, for example, that a system whose objective involves addressing the health care needs of its population must use an HRH planning method that estimates HRH requirements as a function of population health measures so that resources can be planned in accordance with levels of—and potential changes in—the population’s needs for health care. Resources are then allocated between populations based on differences in needs between those populations and increased or decreased over time in accordance with increases or decreases in those needs, while also allowing for changes in the way needs are to be met (e.g. using new technologies or different types of health care teams). Although meeting population health care needs is a goal shared by many health care systems, the findings of this review indicate that few countries appear to be using needs-based methods for HRH planning. Instead, those HRH analyses we found appear to be using utilization- or supply-based approaches. For countries such as the United States, where the ability to pay for health care services has traditionally been an important element of access to care, utilization-based approaches may be more relevant in addressing the policy questions facing decision-makers. Although some of these approaches (e.g. [70]) incorporate consideration of how population health needs affect service use, this is done within the context of the prevailing organization of services in the United States and hence are unlikely to represent how needs for care would be served in the absence of payment at the point of delivery or via private insurance plans as a means of using care. For countries where meeting population health care needs is a primary objective, however, failure to plan for HRH according to those needs means planning not to meet that system objective. Moreover, the use of supply- or utilization-based approaches will perpetuate and exacerbate existing inefficiencies and inequalities in these systems ([2, 113, 114]).
  Needs-based approaches to HRH and health system planning are not new. Over 40 years ago, the WHO [115], for example, outlined a history of them dating back to at least the 1930s in the United States, also noting their widespread use in what are now former Soviet states. A more detailed description was later provided by Hall [116], and subsequently, such approaches were also described in the 1980s and 1990s in the United Kingdom [117, 118] and Canada [119–122]. Several more recent examples from Australia [53–56], Canada [2, 27, 57, 58, 104], and New Zealand [29, 59–61] were identified during this review.
2. ᅟ
  - HRH requirements are derived from service requirements, and
  - Those service requirements are aligned with system objectives.
  Requirements for HRH are a manifestation of requirements for the services they provide. Hence, estimates of HRH requirements must be derived from estimates of the requirements for those services. This makes it possible to consider and plan for potential future changes in the way services are delivered resulting from new technologies, changes in scopes of practice, and so on. The results of this review show, however, that HRH planning approaches that cannot account for such changes—such as the use of provider-population ratios—remain prevalent. This is unfortunate given that the major limitations of such approaches have been extensively documented for decades. For example, the WHO noted over 40 years ago that such approaches may be appropriate in,
  …a country, whatever its stage of development, where professional judgement, backed by utilization studies, suggests that the current patterns of the organizations and delivery of health services [through] existing ratios do not present any anomalies or problems. Unfortunately, such situations are rare, and in any case this static view of society implies that the present situation cannot be significantly improved upon in the foreseeable future.
  The exclusive use of health manpower: population ratios for the estimation of future health manpower requirements is increasingly difficult to justify in the developed countries where, depending on the specific characteristics of the health system, other and more refined criteria can be used to improve the country’s capacity to provide health care. [115].
  Other analyses found in this review use methods based on historical population-utilization ratios without any consideration of whether those ratios are consistent with the objectives of the health care system in question. The use of these methods for planning future services—and hence HRH—perpetuates any current patterns of inappropriate use such as over-reliance on emergency departments instead of primary health care or inadequate access to (and therefore use of) services by disadvantaged populations. The needs-based approaches from Australia, Canada, and New Zealand included in this review do not have this limitation. In addition, some ‘needs-informed’ but ultimately utilization-based approaches, such as those described by Dall and colleagues [70] and Gallagher and colleagues [86], are designed to explicitly account for potential changes to service provision as a consequence of, for example, changes in health care legislation or practice models.
3. The approach considers HRH requirements in the context of production functions for health services (i.e. dependent upon the availability or use of other inputs to service production).
  Although the availability of HRH is important to the delivery of health care services, other types of human and non-human resources, such as facilities, equipment, and medications, are also necessary. Effective health systems planning approaches must recognize this dependency by considering how the availability (or lack thereof) of (a) other HRH and (b) non-human resources may affect their collective production of health care services, including the potential for substitution of one type of resource for another. For example, the availability of operating theatre nurses or operating theatres may impact on the volume of surgeries that surgeons can perform, even if the number of surgeons and the hours they work remain the same. The review found several examples of approaches that explicitly incorporated this potential for different types of HRH (e.g. [29, 53, 123]). However, although documents sometimes acknowledged the influence of the availability of non-human resources on HRH requirements, the review found no analyses that directly incorporated this relationship.
4. The approach explicitly considers the role and determinants of productivity (i.e. units of service per hour of work).
  In order to translate health care service requirements into HRH requirements, HRH planners must consider the rate at which different types of HRH are able to provide those services per unit time—i.e. their productivity—under a given set of circumstances. In addition to the needs-based approaches referred to above, numerous other analyses found by the review (e.g. [25, 96, 124, 125]) explicitly included productivity as part of their calculations. Although the contexts in which productivity was considered varied widely across these documents, they generally showed that projections regarding the future HRH situation are highly sensitive to even small changes to HRH productivity.
5. HRH supply is measured in terms of time devoted to service delivery (i.e. flow generated by a stock of HRH) as opposed to focusing only on the HRH stock (numbers of HRH).
  The availability of health care services is determined by a number of factors in addition to the raw ‘stock’ or head count of different types of HRH available to provide them. Analyses in many countries have shown how changes or differences in these factors can have profound effects on the effective supply of HRH (e.g. [30, 66, 68]), and most of the HRH supply analyses found through the review considered at least one of these, most frequently hours worked. Several analyses (e.g. [96, 126, 127]), however, did not take any of these factors into account and instead estimated HRH supply based solely on head counts.
6. The approach considers the determinants of flow (e.g. hours worked) and stock (entries/exits) as policy variables.
  The factors that determine the stock and flow of HRH supply, such as the amount of time spent providing patient care (activity levels), and the proportion of licensed HRH who are actively practising (participation levels), are sensitive—to varying degrees—to HRH policies such as education and payment models. Most of the analyses of HRH supply found through the review reflected this situation; in some cases, such factors were the primary focus of the analyses (e.g. [72, 78, 82]).
7. The approach considers the following:
  - The cost implications of HRH plans and
  - The extent to which HRH plans are aligned with health system financial planning

Essential to determining the relative appropriateness of any potential HRH policy is an understanding of its financial implications in the broader context of the jurisdictional fiscal situation. Although many of the documents included in the review acknowledged this point, comparatively few (e.g. [16, 76, 86, 128]) explicitly incorporated financial considerations into their analyses.

**Fig. 7:**
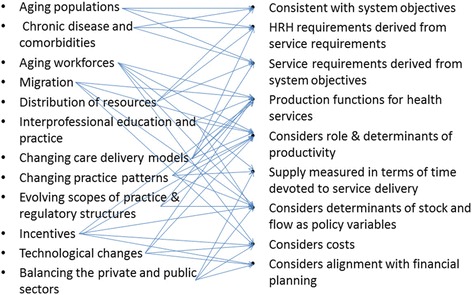
Map of planning inputs to planning criteria

Figure 7 provides a summary illustration of how the various inputs into HRH planning that emerged across the reviewed documents would be addressed by an approach meeting these individual criteria.

Although over 200 documents discussing the HRH situations in the included countries were reviewed as part of this study, it appears that none of these approaches to HRH planning meets all seven of the above criteria. However, several of the approaches described above meet all but one. The needs-based approaches described in Australia [53–56], Canada [57, 58], and New Zealand [29, 59–61] each identify specific service requirements based on population health needs and translate these into HRH requirements based on information on scopes of practice and standards of care delivery. However, the Australian and New Zealand approaches do not appear to explicitly consider the role and determinants of productivity, while the Canadian approach does not include considerations of cost implications. Further, as noted above, none of the identified approaches appears to account for the impact of non-human resources on HRH requirements.

An example of a methodology for projecting HRH requirements in the included countries, then, could build on these needs-based approaches. This would require augmenting them in two ways. First, the Canadian approach would need additional features to include consideration of cost implications, and the Australian and New Zealand approaches would need to be further developed to include consideration of the role and determinants of productivity. Second, both approaches would need further enhancements to include consideration of the impacts of the availability of non-human resources.

The second of these two improvements is evidently the more difficult to achieve, as no HRH planning approaches found through this review included consideration of the impacts of non-human resources. The first of these improvements, however, has already begun. An analytical framework for such an approach has been developed by a team from Canada, Australia, and New Zealand [129] that extends existing needs-based methods to include consideration of the implications of HRH and other health system policies on the system’s fiscal and socio-political sustainability. This framework has not yet been applied and was not included in the review because it was published after its specified time frame. Similar international collaborations may be one way of encouraging the continued advancement of HRH planning methods so that in the future they are also able to account for the impacts of non-human resources.

## Conclusions

This review sought to synthesize the findings of the past decade of published research on HRH requirements and labour market dynamics in high-income OECD countries. Although over 200 documents pertaining to these topics were reviewed in detail, collectively, they do not include sufficient information to provide a clear picture of the expected future HRH situation in these countries. At best, it can be said that the HRH supply in these countries is generally expected to grow. Because different analyses reach different conclusions about future HRH requirements in these countries, it is not clear whether that growth will be adequate to meet health care system objectives in the future. Although most analyses suggest that the numbers of physicians and nurses required in the included countries are likely to increase in the future, this view varies across analyses depending on the methods and assumptions used. Further, most analyses for professions other than nurses and physicians suggest that the numbers required are likely to decrease in the future. The implications of these projected respective surpluses and shortages in terms of meeting health care system objectives are not clear.

Several recurring themes regarding factors of importance in HRH planning were evident across the documents reviewed. These included the following: aging populations and health workforces; changes in disease patterns, models of care delivery, scopes of practice, regulatory structures, and technologies in health care; migration; incentives; data quality; the distribution of resources; interprofessional education and practice; stakeholder engagement; and balancing the public and private sectors. They also included important inconsistencies in the use of key HRH planning terms, which in turn affected the choice of methods in different documents for HRH planning.

Different approaches to HRH planning will be appropriate for different jurisdictions depending on their respective contexts and the objectives of their health care systems and hence the policy questions being faced. Based on these objectives, methods need to be adopted that produce relevant answers for the precise HRH questions facing policymakers in their particular contexts. The results of this review suggest, however, that rarely have explicit policy questions been identified to guide HRH research methods and analyses; instead, available methods have been adopted with policy ‘interests’ (as distinct from questions) made to fit these methods.

In an attempt to inform improvements to this situation, seven criteria for identifying an HRH planning approach appropriate to a given jurisdiction have been presented. Although none of the approaches found through the review met all of these criteria, several—from Australia, Canada, and New Zealand—met all but one. Examples of other approaches which met each individual criterion have also been identified so that planners can explore different options depending on the relative importance of these criteria given their respective circumstances and health system objectives.

Policymakers seeking to act on this evidence may consider multiple actions in the short term that may have short-, medium-, and long-term rewards. These include the following:

1. Identifying and engaging with the relevant HRH stakeholders in their respective jurisdictions to review and discuss the findings of this review and their implications for HRH planning in that jurisdiction. This may include consideration of the degrees to which
  - The criteria for HRH planning approaches identified here are relevant for their jurisdiction,
  - HRH planning approaches currently in use in their jurisdiction are consistent with those criteria, and
  - HRH planning approaches currently in use in their jurisdiction could be improved by incorporating elements from others identified through this review.
2. Assessing the degree to which available data are adequate to inform HRH planning in that jurisdiction. Concerns about data are as long-standing as the study of HRH planning itself [115]. However, as has been noted elsewhere (e.g. [2]), problems with data are not avoided by relying on conceptually invalid models that ignore fundamental health care system objectives such as meeting population health needs by failing to incorporate measures of these or cannot account for potential future changes in factors such as productivity. It is better to base plans on appropriate concepts imperfectly measured than on inappropriate concepts that can be easily measured. If a particular jurisdiction’s HRH stakeholders deemed the data available to them inadequate to fully inform planning, then investments should be made in improving the quality of the available data rather than in further entrenching the use of intrinsically flawed models. To that end, the identification and assessment of the data required to inform HRH planning should be based on the question of how many of what type of HRH are required to perform what services, for whom, and under what circumstances [2].
3. Items (1) and (2) above must be repeated on a regular basis as part of an iterative process for improving the alignment of HRH planning inputs and processes with the objectives of the jurisdiction’s health care system.

## Additional files

Additional file 1: List of websites searched. (DOC 52 kb)
Additional file 2: Data extraction tool for peer- and non-peer-reviewed literature. (DOC 32 kb)
