# Supplementary material for: A synthesis of recent analyses of human resources for health requirements and labour market dynamics in high-income OECD countries
Source: Hum Resour Health. 2016 Sep 29;14:59. doi: 10.1186/s12960-016-0155-2 (PMC5043532; doi:10.1186/s12960-016-0155-2)
Supplement: Additional file 1: — List of websites searched. (DOC 52 kb) [file 12960_2016_155_MOESM1_ESM.doc]

# List of Websites Searched

**International:**

- - GHWA <http://www.who.int/workforcealliance/>
  - WHO Health Workforce <http://www.who.int/hrh/resources/en/>
  - OECD <http://www.oecd.org/>
  - World Bank <http://www.worldbank.org/>
- Andean network of Observatories for human resources for health <http://www.observatoriorh.org/andino/?q=taxonomy/term/23>
- European Observatory on Health Systems and Policies <http://www.euro.who.int/en/about-us/partners/observatory>
- WHO EURO Health Evidence Network (HEN)

<http://www.euro.who.int/en/data-and-evidence/evidence-informed-policy-making/health-evidence-network-hen>

- The Health Systems and Policy Monitor

<http://www.hspm.org/mainpage.aspx>

- European Commission on Public Health, health workforce <http://ec.europa.eu/health/workforce/policy/index_en.htm>
- Joint Action on Health Workforce Planning and Forecasting <http://www.euhwforce.eu/>
  - HRH Global Resource Centre <http://www.hrhresourcecenter.org/>
  - KIT <http://www.kit.nl/kit/en/>
  - GIZ <http://www.giz.de/en/html/index.html>
  - Health Cluster EU [http://healthclusternet.eu](http://healthclusternet.eu/)
  - WHO Collaborating Centers focusing on HRH
    - University of Western Cape <http://www.uwc.ac.za/Faculties/CHS/soph/Pages/WHO-Collaborating-Center-.aspx>
    - University of Illinois at Rockford [http://ncrhp.uic.edu/index.cfm?id=1031&b=1003&page=World%20Health%20Organization%20%28WHO%29%20Collaborating%20Centre](http://ncrhp.uic.edu/index.cfm?id=1031&b=1003&page=World Health Organization (WHO) Collaborating Centre)
    - McMaster University <http://nursing.mcmaster.ca/WHO_collaborating_centre.html>
- WHO Collaborating Center on Health Workforce Policy and Planning - <http://whoccworkforce.ihmt.unl.pt/>

**Country-specific:**

- - Australia
    - Department of Health <http://www.health.gov.au/internet/main/publishing.nsf/Content/Health+Workforce-2>
    - Health Workforce Australia <https://www.hwa.gov.au/>
    - Australia Institute of Health and Welfare <http://www.aihw.gov.au/>
  - Austria
    - Minstiry of Health <http://bmg.gv.at/home/Schwerpunkte/Gesundheitssystem_Qualitaetssicherung/>
    - Public Health Portal <https://www.gesundheit.gv.at/Portal.Node/ghp/public>
  - Belgium
    - Federal Ministry of Health (planning commission) [http://www.health.belgium.be/eportal/Healthcare/Consultativebodies/Planningcommission/index.htm#.VT5tVq3BzGc](http://www.health.belgium.be/eportal/Healthcare/Consultativebodies/Planningcommission/index.htm#.VT5tVq3BzGc)
  - Canada
    - Health Canada (human resources strategy) <http://www.hc-sc.gc.ca/hcs-sss/hhr-rhs/strateg/index-eng.php>
    - CHHRN <http://www.hhr-rhs.ca/>
  - Chile
    - Ministry of Health <http://desal.minsal.cl/>
  - Czech Republic
    - Ministry of Health <http://www.mzcr.cz/Cizinci/>
  - Denmark
    - Health and Medicines Authority <http://sundhedsstyrelsen.dk/en>
  - Estonia
    - National Institute for Health Development <http://www.tai.ee/en/publications/publications>
    - Ministry of Social Affairs <http://www.sm.ee/et>
  - Finland
    - Government VATT Institute for Economic Research <http://www.vatt.fi/en/>
    - National Supervisory Authority for Welfare and Health <http://www.valvira.fi/en/>
    - National Institute for Health and Welfare <https://www.thl.fi/en/web/thlfi-en/publications>
  - France
    - Ministry of Health, Social Affairs and Women’s Rights <http://www.sante.gouv.fr/les-reseaux-de-sante.html>
    - Institute for Research and Information in Health Economics <http://www.irdes.fr/english/home.html>
  - Germany
    - Federal Ministry of Health <http://www.bmg.bund.de/en.html>
    - National Statistic Bureau <https://www.destatis.de/DE/Startseite.html>
  - Greece
    - Ministry of Health and Social Solidarity <http://www.moh.gov.gr/>
  - Iceland
    - Directorate of Health <http://www.landlaeknir.is/english/>
  - Ireland
    - Expert Group on Future Skills Need <http://www.skillsireland.ie/>
    - Department of Health (health services executive) <http://www.hse.ie/eng/>
  - Israel
    - Taub Center for Social Policy Studies in Israel <http://taubcenter.org.il/>
    - Central Bureau of Statistics <http://www1.cbs.gov.il/reader/cw_usr_view_SHTML?ID=590>
    - Ministry of Health <http://www.health.gov.il/Services/Publications/Pages/PublicationsSearch.aspx>
  - Italy
    - Ministry of Health <http://www.salute.gov.it/>
  - Japan
    - Ministry of Health, Labour and Welfare <http://www.mhlw.go.jp/english/>
  - Luxembourg
    - Ministry of Health (health information portal) <http://www.sante.public.lu/fr/index.html>
  - Netherlands
    - NIVEL <http://www.nivel.nl/en>
    - Advisory Committee on Medical Manpower Planning <http://www.capaciteitsorgaan.nl/Publicaties/tabid/68/language/en-US/Default.aspx>
  - New Zealand
    - Health Workforce New Zealand <http://healthworkforce.health.govt.nz/>
    - Ministry of Health <http://www.health.govt.nz/our-work/health-workforce/workforce-service-forecasts>
  - Norway
    - Government of Norway <https://www.regjeringen.no/en/id4/>
    - Directorate of Health <https://helsedirektoratet.no/English>
    - Statistics Norway <http://www.ssb.no/en/>
  - Poland
    - Ministry of Health http://www.mz.gov.pl/wwwmz/index?ml=en
  - Portugal
    - Ministry of Health <http://www.portugal.gov.pt/en/the-ministries/ministry-of-health.aspx>
    - Health Information Portal <http://www.min-saude.pt/portal>
  - Slovakia
    - Ministry of Health <http://www.health.gov.sk/Titulka>
  - Slovenia
    - Ministry of Health <http://www.mz.gov.si/en/>
  - South Korea
    - Ministry of Health and Social Welfare <http://english.mw.go.kr/front_eng/index.jsp>
  - Spain
    - Ministry of Health <http://www.msssi.gob.es/en/home.htm>
  - Sweden
    - Ministry of Health and Social Affairs <http://www.government.se/sb/d/2061>
    - National Board of Health and Welfare <http://www.socialstyrelsen.se/english>
  - Switzerland
    - Swiss Health Observatory <http://www.obsan.admin.ch/bfs/obsan/en/index/01.html>
  - UK
    - Centre for Workforce Intelligence <http://www.cfwi.org.uk/>
    - Department of Health [https://www.gov.uk/government/publications?departments[]=department-of-health](https://www.gov.uk/government/publications?departments%5B%5D=department-of-health)
    - Health Education England <https://hee.nhs.uk/>
    - Northern Ireland Department of Health, Social Services and Public Safety (workforce planning unity) <http://www.dhsspsni.gov.uk/wpu-planning>
    - The Scottish Government <http://www.gov.scot/Publications> /
    - NHS Wales <http://www.wales.nhs.uk/>
    - Wales National Leadership and Innovation Agency <http://www.nliah.wales.nhs.uk/>
  - USA
    - HRSA National Center for Health Workforce Analysis <http://bhpr.hrsa.gov/healthworkforce/>
    - Centre for Health Workforce Studies <http://depts.washington.edu/uwchws/chws-publications.php>
    - Association of School and Programs of Public Health <http://www.aspph.org/>
    - Association of American Medical Colleges <https://www.aamc.org/data/workforce/reports/>
