# Supplementary material for: A synthesis of recent analyses of human resources for health requirements and labour market dynamics in high-income OECD countries
Source: Hum Resour Health. 2016 Sep 29;14:59. doi: 10.1186/s12960-016-0155-2 (PMC5043532; doi:10.1186/s12960-016-0155-2)
Supplement: Additional file 2: — Data extraction tool for peer- and non-peer-reviewed literature. (DOC 32 kb) [file 12960_2016_155_MOESM2_ESM.doc]

**Data extraction tool for peer and non-peer reviewed literature**

| **Authors** | **Title** | **Year of publication** | **Country(s) or jurisdiction(s) included** | **Analysis** | | **Professions included** | **Determinants of supply** | **Determinants of requirements** | **Explicitly considers:** | | | **Assumptions described** | **Trends/issues identified** | **Conclusion reached** | **Other remarkable features** | **Comments** |
| --- | --- | --- | --- | --- | --- | --- | --- | --- | --- | --- | --- | --- | --- | --- | --- | --- |
|  |  |  |  | **Start year** | **End year** |  |  |  | **Population health needs** | **Planned service levels by different levels of need** | **Provider productivity** |  |  |  |  |  |
|  |  |  |  |  |  |  |  |  |  |  |  |  |  |  |  |  |
|  |  |  |  |  |  |  |  |  |  |  |  |  |  |  |  |  |
|  |  |  |  |  |  |  |  |  |  |  |  |  |  |  |  |  |
